# Factors associated with dual sensory impairment in older persons attending the Geriatric Centre in Southwest Nigeria

**DOI:** 10.4314/gmj.v58i2.3

**Published:** 2024-06

**Authors:** Abiola O Obadare, Lawrence A Adebusoye, Eniola O Cadmus

**Affiliations:** 1 Department of Family Medicine, University College Hospital, Ibadan, Nigeria; 2 Chief Tony Anenih Geriatric Centre, University College Hospital, Ibadan, Nigeria and Department of Community Medicine, College of Medicine, University of Ibadan, Nigeria

**Keywords:** Elderly, Hearing Impairment, Visual impairment, Dual sensory impairment, Functional ability, Activity of Daily Living, Quality of Life

## Abstract

**Objective:**

This study examined the prevalence of hearing impairment, visual impairment and Dual Sensory Impairment (DSI) and the risk factors among older persons

**Design:**

A Cross-sectional study where respondents were recruited by systematic random sampling.

**Setting:**

A tertiary institution at the Geriatric Centre, University College Hospital, Ibadan, Nigeria.

**Participants:**

A total of 388 older persons aged more than 60 years were recruited

**Interventions:**

A semi-structured pretested questionnaire was used over three months.

**Main outcome measures:**

Association between ageing, low income, poor quality of life, functional disability, and dual sensory impairment.

**Results:**

The mean age of the respondents was 70.2±6.3 years. The point prevalence of HI, VI and DSI were 14.9%, 8.0%, and 1.5% respectively. On logistic regression analysis, the most significant factors associated with HI were having no formal education OR=2.564(1.091-6.024) and previous hospital admission OR=3.473(1.856-6.499), for VI; increasing age OR=1.080(1.022-1.141) and poor income OR=2.941(1.263-6.897) and DSI; increasing age OR=1.224(1.054-1.421).

**Conclusion:**

Few (1.5%) older adults experienced DSI in our setting. The association between sensory impairments, age, and socioeconomic factors of poor education and income suggests the need for visual and hearing screening in older adults, particularly those with medical and socioeconomic issues, for early detection.

**Funding:**

None declared

## Introduction

The world's population is ageing due to an increasing life expectancy and a decreasing birth rate. All countries, including Nigeria, are experiencing this growth in the number and proportion of older persons.^1^,^2^ The World Health Organization (WHO) describes older adults as 65 years and above. Still, the United Nations describes people aged 60 and above as older because of the low life expectancies in low and medium-income countries (LMICs) such as Nigeria.^1^

The ageing process affects all the organs of the human body,^3^, and research has shown that sensory impairment, particularly hearing and vision deficits, is a usual health problem in older individuals and could occur or increase due to disease or the ageing process.^4^,^5^

The quality of life (QoL) in older persons is reduced by the presence of chronic diseases such as visual impairment, hearing impairment, hypertension, diabetes, chronic back pain, and dementia amongst others.

Dual sensory impairment (DSI), also known as deafblindness or dual sensory loss, is a combination of vision and hearing impairment that interferes with the person's ability to acquire information and communicate with others.^5^ It was found to be associated with poor QoL and an increased risk of mortality among older people.^6^ Some studies in the developed world have shown the deleterious effects of DSI on the QoL of older persons,^4^,^6^ however there has been a dearth of such studies among older Nigerians.^3^ The number of older persons with DSI is expected to increase as a result of the ageing population yet little is known about it.^5^,^7^

Hearing impairment in older people is associated with communication difficulties, depression, social isolation, and poor self-esteem, leading to serious psychosocial and functional problems.^4^ Age-related hearing loss impairs QoL and relationships and increases dependence on community and informal supports.^8^ Even though hearing impairment has been found to contribute to the risk of functional deficit in those with DSI, the effect on disability and QoL remains an under-researched area.^9^

Visual impairment limits independence, reduces the QoL and increases the use of care services and the risk of death.^8^ While each sensory loss has unique detrimental effects on QoL, and together, they present a severe effect^10^

Globally, the estimated number of older individuals reporting the presence of DSI varied considerably.^10^ The early detection and active treatment of sensory impairment in older persons are rarely performed due to the tendency to attribute sensory impairment to the natural ageing process (ageism).^4^ Identified factors associated with DSI in literature were age, functional disability in activities of daily living, cognitive impairment and QoL.^11^

The management of DSI requires a multi-disciplinary approach across vision, hearing, mental health, and chronic illness services in referral, assessment, and rehabilitation.^12^ It is within the purview of healthcare workers to provide good integration of services across these professions. Studies focusing specifically on sensory loss in older persons must inform clinical practice so that DSI, associated factors and its impact can be identified and promptly managed, leading to improved QoL for the older population.^10^

This study aimed to assess the prevalence of hearing impairment (HI), visual impairment (VI), and DSI among older patients attending the Geriatric Centre in the University College Hospital (UCH), Ibadan, Nigeria, and associated factors. The findings are expected to help scale up primary health care services regarding diagnosing and managing elderly patients with DSI.

## Methods

### Study Site

This study was conducted at the Chief Tony Anenih Geriatric Centre (CTAGC) of the University College Hospital, Ibadan. Ibadan is the capital of Oyo state, situated in the Southwestern region of Nigeria, West Africa. The University College Hospital (UCH) was established in Ibadan in 1957. It is the first tertiary hospital in the West African sub-region. It has a capacity of 850 beds with various specialty units. The CTAGC, UCH manages acutely and chronically ill older patients and has facilities for in-patient admission. The CTAGC focuses on providing primary care to older patients at the first point of contact.

### Study Population

The study was a cross-sectional study. Consenting patients attending the Geriatric clinic were recruited by systematic random sampling. Participants aged 60 years and above were recruited consecutively between January and March 2022 from the CTAGC clinics, UCH. The inclusion criteria were older people aged ≥60 who consented to participate in the study. Participants who were acutely ill or did not consent to the study were excluded.

### Study Procedure

All patients gave informed consent. The participants were interviewed using a semi-structured pretested questionnaire, and demographic data, such as respondents' age, gender, years of education, occupational status, and place of domicile, were obtained.

### Study instruments

#### Assessment of Dual Sensory Impairment

All consenting older patients who presented consecutively to the geriatric clinic during the study will be assessed for DSI using the Hearing Handicap Inventory for the Elderly Screening (HHIE-S) and Snellen's chart. All older patients found to have impairment in both the hearing and visual assessments are considered to have DSI.

The HHIE-S is a subjective questionnaire for the assessment of hearing impairment. This questionnaire consists of 10 questions designed to measure the emotional and social level of the elderly with hearing loss. Assessment of the yes is (4 points), sometimes (2 points), or no (0 points) for each question. The minimum score that can be achieved is 0, and the highest score that can be achieved is 40. The score is interpreted as no auditory handicap (0 to 8 points), mild to moderate auditory handicap (10 to 24 points), or significant auditory handicap (above 24 points).^13^,^14^

Despite the high prevalence of hearing disorders in the elderly, in-depth investigation, such as Pure Tone Audiometry, is still generally restricted to medical evaluations. Thus, screening for HI among the elderly using a standardised questionnaire such as an HHIE-S is more suitable for identifying more disabling hearing loss and is a useful tool in primary care.^15^

Snellen's chart was used to assess the visual impairment (VI) based on The International Classification of Diseases -10 which classified VI into Mild or no VI (category 0) for visual acuity (VA) ≥ 6/18, moderate VI (category 1) for VA ≤ 6/18 to ≥ 6/60, severe VI (category 2) for VA≤ 6/60 to ≥ 3/60 and blindness (category 3, 4 and 5) for VA < 1/60 to no light perception.^16^ The chart was placed 6 metres away from the participants.^16^

#### Assessment of Quality of Life

The brief version of the World Health Organization's QoL (WHOQoL- Bref) instrument was used to assess the QoL of the participants. The instrument has 26 items grouped into four major domains, namely, the first 2 items, physical capacity (7 items), psychological well-being (6 items), social relationships (3 items), and environment (8 items). Each item is measured on a 5-point scale (1–5). A global score was obtained by combining all item scores with a maximum obtainable score of 130. Scores below 70% of the maximum score (less than 91) were considered indicative of “poor QOL.” Scores from 70% and above were considered as indicative of “good QOL.^17^,^18^

Functional abilities were assessed using the Katz Index of Basic Activities of Daily Living (BADL). A score of 6 indicates full function, 4 indicates moderate impairment, and 2 or less indicates severe functional impairment.^19^ It has been used in Nigeria.^20^

### Ethical considerations

Informed consent was obtained from each respondent or proxy, and ethical approval for the study was obtained from the University of Ibadan/University College Hospital Institutional Review Board (UI/EC/21/0039).

### Data analysis

The administered questionnaires were checked, sorted and coded serially on each study day. Data entering, cleaning and analysis were done using SSPS (version 27). Descriptive statistics were used to assess the sociodemographic characteristics of the respondents. Appropriate charts were used to illustrate categorical variables. The Chi-square test was used for the categorical variables. Logistic regression analysis was used to explore significant variables with HI, VI and DSI. The p-value of significance was set at ≤ 0.05.

## Results

There were 388 respondents with a mean ± SD age of 70.2 ± 6.3 years; the majority, 268 (69.07%) were females. The male respondents were mostly married (90.9%), had formal education (62.5%), earned above the 30,000 Naira Nigerian minimum monthly wage (81.7%), experienced good quality of life (88.0%), and were functionally independent (97.5%). While the majority of the female respondents were not engaged in occupational activities (86.9%), physically active (99.3%), lived with their children/grandchildren (51.1%), had no previous hospital admission (56.0%), and all self-rated their health as good (100.0%). (Table 1).

**Table 1:** Baseline characteristics of the respondents

	Males = 120 n (%)	Females = 268 n (%)	Total = 388 N (%)
**Age (Years)**			
**60 – 69**	62 (51.6)	138 (51.5)	200 (51.6)
**70 – 79**	47 (39.2)	106 (39.5)	153 (39.4)
**≥80**	11 (9.2)	24 (9.0)	35 (9.0)
**Marital status**			
**Married**	109 (90.9)	139 (51.9)	248 (63.9)
**Widowed**	10 (8.3)	122 (45.5)	132 (34.0)
**Separated/divorced**	1 (0.8)	7 (2.6)	8 (2.1)
**Highest Educational attainment**			
**None**	5 (4.2)	27 (10.1)	32 (8.3)
**Primary**	16 (13.3)	47 (17.5)	63 (16.2)
**Secondary**	24 (20.0)	71 (26.5)	95 (24.5)
**Tertiary**	75 (62.5)	123 (45.9)	198 (51.0)
**Monthly income (Naira)**			
**≤ 30,000**	22 (18.3)	109 (40.7)	131 (33.8)
**> 30,000**	98 (81.7)	159 (59.3)	257 (66.2)
**Occupational activities**			
**Engaged in occupational activities**	21 (17.5)	35 (13.1)	56 (14.4)
**Not engaged in occupational activities**	99 (82.5)	233 (86.9)	332 (85.6)
**Level of physical activities**			
**Not active**	1 (0.8)	2 (0.7)	3 (0.8)
**Active**	119 (99.2)	266 (99.3)	385 (99.2)
**Living arrangement**			
**Alone**	2 (1.7)	6 (2.3)	8 (2.1)
**With spouse**	97 (80.8)	118 (44.0)	215 (55.4)
**With Children/Grandchildren**	19 (15.8)	137 (51.1)	156 (40.2)
**Friends/Relatives**	2 (1.7)	7 (2.6)	9 (2.3)
**Previous hospital admission**			
**Yes**	54 (45.0)	118 (44.0)	172 (44.3)
**No**	66 (55.0)	150 (56.0)	216 (55.7)
**Quality of Life**			
**Poor**	24 (20.0)	64 (23.9)	88 (22.7)
**Good**	96 (88.0)	204 (76.1)	300 (77.3)
**Self-rated Health**			
**Poor**	1 (0.8)	0 (0.0)	1 (0.3)
**Good**	119 (99.2)	268 (100.0)	387 (99.7)
**Took Alcohol**			
**Yes**	5 (4.2)	1 (0.4)	6 (1.5)
**No**	115 (95.8)	267 (99.6)	382 (98.5)
**Took Tobacco**			
**Yes**	8 (6.7)	1 (0.4)	9 (2.3)
**No**	112 (93.3)	267 (99.6)	379 (97.7)
**Functional ability**			
**Dependent**	3 (2.5)	12 (4.5)	15 (3.9)
**Independent**	117 (97.5)	256 (95.5)	373 (96.1)

### Prevalence of sensory impairment

The point prevalence of hearing, visual, and dual sensory impairment was 14.9%, 8.0% and 1.5%, respectively. Table 2 shows the factors associated with hearing impairment. Increasing age (p = 0.015), previous hospital admission (p <0.0001), and having no formal education (p = 0.007) were significantly associated with hearing impairment. In addition, hearing impairment was significantly associated with poor quality of life (p = 0.020).

**Table 2:** Factors associated with hearing impairment

Variable	Hearing Impairment			
	Yes = 58 n (%)	No = 330 n (%)	χ^2^	p
**Sex**				
**Male**	20 (16.7)	100(83.3)	0.403	0.525
**Female**	38 (14.2)	230(85.8)		
**Age Group (Years)**				
**60 – 69**	28(14.0)	172 86.0)	8.389	0.015*
**70 – 79**	19 (12.4)	134(87.6)		
**≥80**	11 (31.4)	24 (68.6)		
**Marital Status**				
**Currently Married**	37 (14.9)	211 (85.1)	0.000	0.983
**Not Married**	21 (15.0)	119 85.0)		
**Educational Status**				
**Had no formal**	10 (31.2)	22 (68.8)	7.290	0.007*
**Had formal**	48 (13.5)	308 86.5)		
**Income/month**				
**≤ 30,000 Naira**	21 (16.0)	110(84.0)	0.182	0.670
**> 30,000 Naira**	37 (14.4)	220(85.6)		
**Occupational activities**				
**Not engaged in occupational activities**	53 (16.0)	279 (84.0)	1.865	0.172
**Engaged in occupational activities**	5 (8.9)	51 (91.1)		
**Level of physical activities**				
**Not Active**	44 (17.1)	214(82.9)	2.686	0.101
**Active**	14 (10.8)	116(89.2)		
**Living arrangement**				
**Alone**	2 (25.0)	6 (75.0)	0.649	0.420
**With Others**	56 (14.7)	324(85.3)		
**Previous hospital admission**				
**Yes**	41 (23.8)	131(76.2)	19.200	<0.0001*
**No**	17 (7.9)	199(92.1)		
**Quality of Life**				
**Poor**	20 (22.7)	68 (77.3)	5.417	0.020*
**Good**	38 (12.7)	262(87.3)		
**Took Alcohol**				
**Yes**	1 (16.7)	5 (83.3)		0.905^f^
**No**	57 (14.9)	325(85.1)		
**Took Tobacco**				
**Yes**	3 (33.3)	6 (66.7)		0.118^f^
**No**	55 (14.5)	324(85.5)		
**Functional ability**				
**Dependent**	4 (26.7)	11 (73.3)		0.194^f^
**Independent**	54 (14.5)	319 85.5)		

* Significant at 5% level of significance

f Fisher's Exact Test

The factors associated with visual impairment are shown in Table 3. Advancing age (p <0.0001) and earning below the Nigerian minimum wage of 30,000 Naira per month (p <0.0001) were significantly associated with visual impairment. Also, there was a statistical association between visual impairment and having poor quality of life (p <0.0001).

**Table 3:** Factors associated with visual impairment

Variable	Visual Impairment			
	Yes = 31 n (%)	No = 357 n (%)	χ^2^	P
**Sex**				
**Male**	9 (7.5)	111 92.5)	0.057	0.812
**Female**	22 (8.2)	246(91.8)		
**Age Group (Years)**				
**60 – 69**	10 (5.0)	190 (95.0)	17.394	<0.0001*
**70 – 79**	12 (7.8)	141 (92.2)		
**≥80**	9(25.7)	26 (74.3)		
**Marital Status**				
**Currently Married**	15 (6.0)	233 (94.0)	3.524	0.061
**Not Married**	16(11.4)	124 (88.6)		
**Educational Status**				
**Had no formal**	4 (12.5)	28 (87.5)	0.965	0.326
**Had formal**	27 (7.6)	329 (92.4)		
**Income/month**				
**≤ 30,000 Naira**	21 (16.0)	110 (84.0)	17.394	<0.0001*
**> 30,000 Naira**	10 (3.9)	247 (96.1)		
**Occupational activities**				
**Not engaged in occupational activities**	29 (8.7)	303 (91.3)	1.738	0.187
**Engaged in occupational activities**	2 (3.6)	54 (96.4)		
**Level of physical activities**				
**Not active**	24 (9.3)	234 (90.7)	1.805	0.179
**Active**	7 (5.4)	123 (94.6)		
**Living arrangement**				
**Alone**	0 (0.0)	8 (100.0)	0.709	0.400
**With Others**	31 (8.7)	349 (91.8)		
**Previous hospital admission**				
**Yes**	12 (7.0)	160 (93.0)	0.431	0.511
**No**	19 (8.8)	197 (91.2)		
**Quality of Life**				
**Poor**	15(17.0)	73 (83.0)	12.696	<0.0001*
**Good**	16 (5.3)	284 (94.7)		
**Took Alcohol**				
**Yes**	0 (0.0)	6 (100.0)		0.467^f^
**No**	31 (8.1)	351 (91.9)		
**Took Tobacco**				
**Yes**	0 (0.0)	9 (100.0)		0.371^f^
**No**	31 (8.2)	348 (91.8)		
**Functional ability**				
**Dependent**	3 (20.0)	12 (80.0)		0.080^f^
**Independent**	28 (7.5)	345 (92.5)		

* Significant at 5% level of significance

f Fisher's Exact Test

The factors associated with dual sensory impairment were increasing age (p <0.0001), being currently not married (p = 0.015), and earning below the Nigerian minimum wage of 30,000 Naira per month (p = 0.001) were significantly associated with dual sensory impairment. Having dual sensory impairment was significantly associated with poor quality of life (p <0.0001) and being dependent in functional ability (p <0.0001) (Table 4).

**Table 4:** Factors associated with dual sensory impairment

Variable	Dual Sensory Impairment			
	Yes = 6 n (%)	No = 382 n (%)	χ^2^	P
**Sex**				
**Male**	0 (0.0)	120(100.0)		0.099^f^
**Female**	6 (2.8)	262 (97.8)		
**Age Group (Years)**				
**60 – 69**	0 (0.0)	200 (100.0)		<0.0001^f^*
**70 – 79**	2 (1.3)	151 (98.7)		
**≥80**	4 (11.4)	31 (88.6)		
**Marital Status**				
**Currently Married**	1 (0.4)	247 (99.6)		0.015^f^*
**Not Married**	5 (3.6)	135 (96.4)		
**Educational Status**				
**Had no formal**	1 (3.1)	31 (96.9)		0.450^f^
**Had formal**	5 (1.4)	351 (98.6)		
**Income/month**				
**≤ 30,000 Naira**	6 (4.6)	125 (95.4)		0.001^f^*
**> 30,000 Naira**	0 (0.0)	257 (100.0)		
**Occupational activities**				
**Not engaged in occupational activities**	6 (1.8)	326 (98.2)		0.311^f^
**Engaged in occupational activities**	0 (0.0)	56 (100.0)		
**Level of physical activities**				
**Not active**	6 (2.3)	252 (97.7)		0.080^f^
**Active**	0 (0.0)	130 (100.0)		
**Living arrangement**				
**Alone**	0 (0.0)	8 (100.0)		0.720^f^
**With Others**	6 (1.6)	374 (98.4)		
**Previous hospital admission**				
**Yes**	3 (1.7)	169 (98.3)		0.778^f^
**No**	3 (1.4)	213 (98.6)		
**Quality of Life**				
**Poor**	5 (5.7)	83 (94.3)		<0.0001^f^*
**Good**	1 (0.3)	299 (99.7)		
**Took Alcohol**				
**Yes**	0 (0.0)	6 (100.0)		0.096^f^
**No**	6 (1.6)	376 (98.4)		
**Took Tobacco**				
**Yes**	0 (0.0)	9 (100.0)		0.704^f^
**No**	6 (1.6)	373 (98.4)		
**Functional ability**				
**Dependent**	2 (13.3)	13 (86.7)		<0.0001^f^*
**Independent**	4 (1.1)	369 (98.9)		

* Significant at 5% level of significance

f Fisher's Exact Test

The logistic regression analysis was conducted for factors that showed significant associations at the bivariate level. For hearing impairment, the most significant factors were having no formal education OR = 2.564 (95% CI: 1.091 - 6.024), p = 0.031 and having had a previous hospital admission OR = 3.473 (95% CI: 1.856 - 6.499), p <0.0001. Increasing age OR = 1.080 (95% CI: 1.022 -1.141), p = 0.006 and earning below the Nigerian minimum wage of 30,000 Naira per month OR = 2.941 (95% CI: 1.263 - 6.897), p = 0.012 were the most significant factors associated with visual impairment. For respondents with dual sensory impairment, increasing age OR = 1.224 (95% CI: 1.054 - 1.421), p = 0.014 was the most significant factor. Table 5,

**Table 5:** Results of multivariate analysis for factors most significantly associated with Hearing Impairment, Visual Impairment, and Dual Sensory Impairment

Variable	B	p-value	Odds Ratio	95% CI for OR	
				Lower	Upper
**HEARING IMPAIRMENT**					
Age (Years)	0.016	0.479	1.017	0.971	1.064
Had poor Quality of Life	0.631	0.054	1.879	0.988	3.571
Had no formal education	0.942	0.031*	2.564	1.091	6.024
Had previous hospital admission	1.245	<0.0001*	3.473	1.856	6.499
Constant	-2.603	0.193	0.074		

**VISUAL IMPAIRMENT**					
Age (Years)	0.077	0.006*	1.080	1.022	1.141
Had Poor Quality of Life	0.781	0.059	2.183	0.969	4.926
≤ 30,000 Naira per month	1.080	0.012*	2.941	1.263	6.897
Constant	-5.834	0.011	0.003		

**DUAL SENSORY IMPAIRMENT**					
Age (Years)	0.200	0.014*	1.221	1.041	1.432
Not currently married	0.005	0.997	1.005	0.078	12.960
Had Poor Quality of Life	-1.723	0.160	0.178	0.016	1.972
≤ 30,000 Naira per month	-16.602	0.994	0.000	0.000	.
Functionally dependent	0.119	0.934	1.126	0.069	18.319
Constant	-1.227	1.000	0.293		

* Significant at 5% level of significance

## Discussion

This study determined the prevalence of dual sensory impairment (DSI), hearing impairment (HI) and visual impairment (VI) and the factors associated with these sensory impairments among older Nigerians. The point prevalence of DSI, HI and VI was 1.5%, 14.9% and 8.0% respectively. A low prevalence of DSI similar to our study was reported among older persons in sub-Saharan African countries like Ghana (2.02%) and South Africa (3.64%) by the World Federation of the Deaf-Blind (WFDB).^21^ In comparison to our report, a higher prevalence of DSI was found among community-dwelling older adults in Malaysia, where a prevalence of DSI (10.5%) was reported.^11^ This disparity may be due to the use of pure tone audiometry compared to the HHIE-S used in this study. However, a lower prevalence of DSI (0.65%) was observed in a population-based prospective cohort study in the UK. This difference could be attributed to the lower cut-off age of 40 years used in the study to define the older population.^22^

The prevalence of HI was higher in our study compared to the survey carried out among older Nigerians, where the prevalence of HI was 6.1%.^9^ Correia *et al.* also reported a lower prevalence (5.3%) among older community-based Americans.^23^ Statement from a cohort study from the UK Biobank reported that over 10% of older adults worldwide had HI.^21^ Hudu *et al.* reported a higher prevalence of VI (18.8%) among older Nigerians compared with our study despite using a low cut-off age of 50 years to define their study population.^24^ Similarly, a higher prevalence was reported in older South Africans (63.6%)^25^ and older Indians (33.1%).^26^ The global differences in the reported prevalence of these sensory impairments could result from the numerous diagnostic tools, clinical assessment, definitive criteria, and other methodological challenges.

This present study found that advancing age was significantly associated with HI, VI and DSI. Virtually all studies reported a relationship between increasing age and sensory impairments.^11^, ^21^-^26^ The prevalence of VI and blindness was found in a South African study to be high among residents in low-income old age homes living in Durban.^25^ Kwon *et al.* reported that older Iranians who had DSI were of a lower economic status (3.9%) compared to those of high socioeconomic status.^4^ In Nigeria, VI was found to be associated with low education, similar to our finding.^27^,^28^ Older respondents without a formal education had 2.5 odds of having HI.

Kwon *et al.* found an association between low educational status and the prevalence of sensory impairments.^4^ Significantly, sensory impairments (HI, VI, and DSI) were associated with poor quality of life (QoL).

Lasisi *et al.*, in the same city as this study, found older people with HI having poor or reported poor QoL.^9^ Most studies in a systematic review by Tseng *et al.* found that HI is significantly associated with poor QoL.^29^ Reducing hearing difficulties by correcting HI has significantly improved the QoL among older South Africans.^30^ In Nigeria, Adigun et al. similarly found poor quality of health in 64.2% of adult patients recruited, whereas more than half of the participants were older patients with visual impairment.^28^

There is a shortage of studies on DSI in Nigeria. Increasing age, low income, poor QoL, and functional disability were factors associated with DSI found in this current study. Few studies in Nigeria that looked into different aspects of QoL in older persons focused mainly on general QoL and QoL associated with some chronic diseases.^9^,^17^,^18^ Still, no studies have been done on the QoL in older individuals with DSI.^9^,^17^,^18^ Mihocek *et al.*, reported that Deaf-blind individuals are mostly dissatisfied with their QoL.^32^ Also, people with DSI experienced worse QoL than those with HI or VI individually.^4^,^29^ Higher mortality (44.0%) has been reported among older people with DSI compared with no sensory impairment.^22^

In our study, a higher proportion of participants with sensory impairments had functional disabilities. In similarity, Lasisi *et al.* found functional disability among 35.4% of community-based older persons in southwest Nigeria.^9^ Older adults with DSI are twice at risk of having a problem with mobility.^4^ DSI affects mainly the mobility domain of the activities of daily living (ADL) in older adults, which negatively affects their QoL.^31^ DSI has been reported as the strongest predictor of functional disability in older persons.^32^

### Limitations

We observe some limitations in our study. The cross-sectional design of this study limits the establishment of a temporal relationship between the variables. Also, the assessment of hearing impairment in this study with HHIE limits the generalizability of the findings to the general populace. Pure tone audiometry is a better assessment of hearing impairment than HHIE.

## Conclusion

This study found that advancing age generally affects sensory impairments in older persons. Modifiable causative factors such as economic and educational status were associated with sensory impairment. Outcome factors like functional disability and poor QoL were more common in older persons with sensory impairment. Thus, routine assessment of sensory impairment, such as DSI, for early diagnosis and interventions in older persons would help alleviate the associated burden. The HHIE-S screening tool for hearing impairment has been recognised as a useful tool that can be used in primary care where pure tone audiometry is considered unavailable, inaccessible and unaffordable.
